# BAP1 Loss Might Be a Predictive Biomarker for Immunotherapy Response in Pleural Mesothelioma

**DOI:** 10.1111/1759-7714.70343

**Published:** 2026-07-01

**Authors:** Reina Imase, Rie Sakakibara, Susumu Kirimura, Hironori Ishibashi, Kenichi Okubo, Yasunari Miyazaki

**Affiliations:** ^1^ Department of Respiratory Medicine Institute of Science Tokyo Tokyo Japan; ^2^ Department of Pathology Institute of Science Tokyo Tokyo Japan; ^3^ Department of Thoracic Surgery Institute of Science Tokyo Tokyo Japan

**Keywords:** BAP1, immune checkpoint inhibitor, immunohistochemistry, immunotherapy, pleural mesothelioma

## Abstract

**Introduction:**

Reliable predictive biomarkers for immune checkpoint inhibitors (ICIs) in pleural mesothelioma (PM) are lacking. Loss of BRCA1‐associated protein 1 (BAP1) is a frequent molecular alteration in PM and may influence the tumor immune microenvironment. We evaluated whether BAP1 loss is associated with clinical outcomes following immunotherapy.

**Methods:**

We retrospectively analyzed 14 patients with PM who were treated with ICIs between April 2014 and May 2024. BAP1 status was assessed by immunohistochemical staining of resected tumor specimens. Patients were categorized into BAP1‐loss and BAP1‐positive groups. Progression‐free survival (PFS), overall survival (OS), response rate (RR), and disease control rate (DCR) were compared.

**Results:**

BAP1 loss was observed in nine patients (64%). Median PFS was significantly longer in the BAP1‐loss group compared with the BAP1‐positive group (5.3 vs. 2.1 months; log‐rank *p* = 0.03). Median OS was also prolonged in the BAP1‐loss group (8.6 vs. 2.1 months; log‐rank *p* = 0.03). RR and DCR were numerically higher among patients with BAP1 loss.

**Conclusion:**

BAP1 loss was associated with improved clinical outcomes following immunotherapy in PM. These findings support that a molecular profile that includes BAP1 deletion may serve as a biomarker for predicting response to immunotherapy in PM.

## Graphical abstract

We evaluated whether BAP1 loss is associated with clinical outcomes following immunotherapy. BAP1 loss shows better progression‐free survival (PFS), but we can't conclude that BAP1 loss is a favorable predictive biomarker. The reason is that BAP1 loss may have a better baseline prognosis, and it is necessary to consider the interactions with other genes.

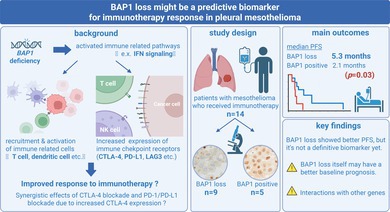

**Abbreviations**
BAP1: BRCA1‐associated protein 1
CR: complete response
DCR: disease control rate
ECOG: Eastern Cooperative Oncology Group
ICI: immune checkpoint inhibitor
IHC: immunohistochemistry
mRECIST: modified response evaluation criteria in solid tumors
OS: overall survival
PD: progressive disease
PFS: Progression free survival
PM: pleural mesothelioma
PR: partial response
PS: performance status
PS‐L1: programmed death‐ligand 1
RR: response rate
SD: stable disease

## 1 Introduction

Pleural mesothelioma (PM) is a rare and aggressive malignancy with a poor prognosis [1]. The CheckMate 743 trial demonstrated that combination therapy with nivolumab plus ipilimumab improved survival compared with chemotherapy in previously untreated patients with PM, establishing this regimen as a new standard first‐line treatment. However, the median overall survival (OS) remains approximately 18 months, with a survival benefit of only 4 months [2]. Furthermore, programmed death‐ligand 1 (PD‐L1) expression does not predict the efficacy of immunotherapy in PM [2, 3, 4, 5, 6], and other reliable predictive biomarkers have yet to be identified.

BRCA1‐associated protein1 (BAP1) is a tumor suppressor gene and one of the most common driver gene alterations, observed in approximately 50% of patients with PM [7]. BAP1 encodes a deubiquitinating enzyme involved in diverse nuclear cellular functions, including the regulation of transcription factors, chromatin remodeling, and DNA repair. In addition, BAP1 is thought to play a crucial role in cytoplasmic apoptosis. Loss‐of‐function alterations in BAP1 are presumed to result in impaired DNA damage repair and accumulation of genomic instability, ultimately contributing to malignant transformation [8].

Several reports indicate that BAP1 loss is associated with favorable prognosis [9, 10, 11, 12, 13]. However, other reports suggest that BAP1 loss is associated with poor prognosis [14]. Furthermore, a meta‐analysis analyzing 698 patients with PM found no difference in prognosis based on BAP1 loss status [15]. Therefore, the association between BAP1 status and prognosis remains controversial. Moreover, only a limited number of studies have evaluated the relationship between BAP1 status and the efficacy of platinum‐based chemotherapy [15, 16, 17], and no consensus has been reached. To date, only one study has examined the relationship between BAP1 status and the efficacy of immunotherapy in PM, reporting no significant difference in progression‐free survival (PFS) between patients with and without BAP1 alteration [16]. In contrast, transcriptomic analyses have shown enrichment of inflammatory response pathways in BAP1‐deficient PM compared with BAP1‐wild‐type tumors. Furthermore, multi‐omics analyses of peritoneal mesothelioma have demonstrated that BAP1 deficiency is correlated with an inflammatory tumor microenvironment [18]. These findings suggest that BAP1 status may serve as a predictive biomarker for the efficacy of immunotherapy. Therefore, we conducted a retrospective study to evaluate whether BAP1 loss is associated with clinical outcomes following immune checkpoint inhibitor (ICI) therapy in patients with PM.

## 2 Methods

### 2.1 Patient Selection and Data Collection

The study population consisted of consecutive patients diagnosed with pleural mesothelioma (PM) between April 2014 and May 2024. From this cohort, we identified and retrospectively analyzed patients that received immunotherapy on or after October 2018. Among patients diagnosed prior to 2018, some received immunotherapy later in their disease course. Data on patient baseline characteristics (age, sex, history of any asbestos exposure, smoking history, Eastern Cooperative Oncology Group (ECOG) performance status (PS)), histological type, treatment history, type of ICI administered, and clinical outcomes were obtained from electronic medical records. ICI regimens were categorized into two groups: nivolumab monotherapy and combination therapy with nivolumab and ipilimumab. BAP1 status was determined based on immunohistochemical analysis, and patients were classified into two groups according to BAP1 expression: BAP1‐loss and BAP1‐positive. The efficacy of immunotherapy was compared between these two groups. Tumor response to immunotherapy was evaluated by pulmonologists using modified Response Evaluation Criteria in Solid Tumors (mRECIST). This evaluation resulted in the classification of the response as complete response (CR), partial response (PR), stable disease (SD), or progressive disease (PD). PFS was defined as the primary endpoint calculated as the interval from the initiation of immunotherapy to the earliest occurrence of documented disease progression or death from any cause. Secondary endpoints included OS, response rate (RR), and disease control rate (DCR). OS was defined as the time from the initiation of immunotherapy to death from any cause or last follow‐up. RR was defined as the proportion of patients achieving CR or PR, and DCR was calculated as the proportion achieving CR, PR, and SD.

### 2.2 Immunohistochemistry

BAP1 protein expression was evaluated by immunohistochemistry (IHC) using 3‐μm‐thick formalin‐fixed, paraffin‐embedded sections obtained from resected or biopsied tumor specimens. For each case, an experienced pulmonary pathologist selected one slide showing the tumor's most representative histology. IHC was conducted using the Leica Bond III automated system (Leica Biosystems Melbourne Pty Ltd., Melbourne, Australia), with BAP1 (mouse monoclonal, clone C‐4, diluted 1:50; Santa Cruz Biotechnology, Dallas, TX) antibody. Nuclear BAP1 expression in tumor cells was assessed by the pathologist. Tumors exhibiting nuclear BAP1 staining in one or more cells were classified as BAP1‐positive, whereas tumors lacking nuclear staining in all tumor cells were classified as BAP1‐loss. We confirmed that vascular endothelium and lymphocytes were BAP1‐positive as positive controls.

### 2.3 Statistical Analysis

Statistical analyses of patient characteristics and responses to immunotherapy were performed using the Mann–Whitney *U* test for continuous variables and the chi‐square test or Fisher's exact test for categorical variables, as appropriate. PFS and OS were analyzed using the Kaplan–Meier curves, and the differences between groups were assessed using the log‐rank test. All statistical analyses were conducted using the Python Software (version 3.14.0).

## 3 Results

### 3.1 Study Flow and Patient Characteristics

**FIGURE 1:**
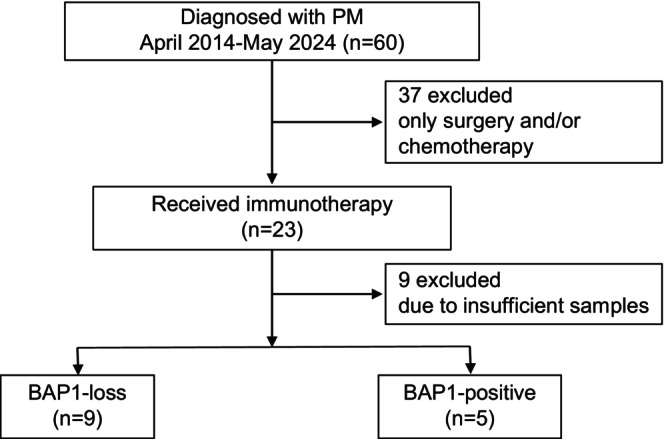
Study flow diagram.

Between April 2014 and May 2024, a total of 60 patients were diagnosed with PM. Among them, 23 patients received immunotherapy on or after October 2018, and 9 patients were excluded because BAP1 status was unavailable. Of the remaining 14 patients who were evaluated for BAP1 status, 9 were classified as BAP1 loss and 5 as BAP1‐positive (Figure 1).

Patient characteristics are shown in Tables 1 and S1. The median age of the entire cohort was 63.5 years, and 90% of patients were male. Epithelioid histology accounted for 71% of cases, and 71% of patients had a history of asbestos exposure, consistent with previous reports [19]. Seven patients received the combination therapy with nivolumab plus ipilimumab, while the remaining seven received nivolumab monotherapy. No significant differences in baseline characteristics were observed between the BAP1‐loss and BAP1‐positive groups.

**TABLE 1:** Comparison of patients characteristics in the BAP1‐loss and BAP1‐positive groups.

	Total (*n* = 14)	BAP1 loss (*n* = 9)	BAP1 positive (*n* = 5)	*p*
Median age (range)	63.5 (45–80)	64 (45–79)	63 (50–80)	0.42
Sex				0.36
Male	13 (93)	9 (100)	4 (80)	
Female	1 (7)	0	1 (20)	
Histological type				0.17
Epithelial type	10 (71)	6 (67)	4 (80)	
Biphasic	3 (21)	3 (33)	0	
Sarcoma type	1 (7)	0	1 (20)	
Asbestos exposure	10 (71)	8 (89)	2 (40)	0.09
Smoking history	11 (79)	7 (78)	4 (80)	0.73
ECOG PS				0.16
0–1	13 (93)	9 (100)	4 (80)	
2–4	1 (7)	0	1 (20)	
Stage				0.45
I–II	10 (71)	7 (78)	3 (60)	
III–IV	4 (29)	2 (22)	2 (40)	
Post surgery	12 (86)	8 (89)	4 (80)	0.65
Pretreatment (including adjuvant therapy)				0.28
Platinum+pemetrexed	13 (87)	9 (100)	4 (80)	
Platinum+gemcitabine	1 (7)	1 (11)	0	
Pemetrexed	1 (7)	0	1 (2)	
ICI				0.58
Ipilimumab+nivolumab	7 (50)	4 (44)	3 (60)	
Nivolumab	7 (50)	5 (56)	2 (40)	

*Note:* Data are presented as number (%) unless otherwise specified. *p* value was calculated using the Mann–Whitney *U* test for continuous variables and chi‐square test or Fisher's exact test for categorical variables, as appropriate.

Abbreviations: ECOG PS, Eastern Cooperative Oncology Group Performance Status; ICI, immune checkpoint inhibitor.

### 3.2 Immunohistochemistry

**FIGURE 2:**
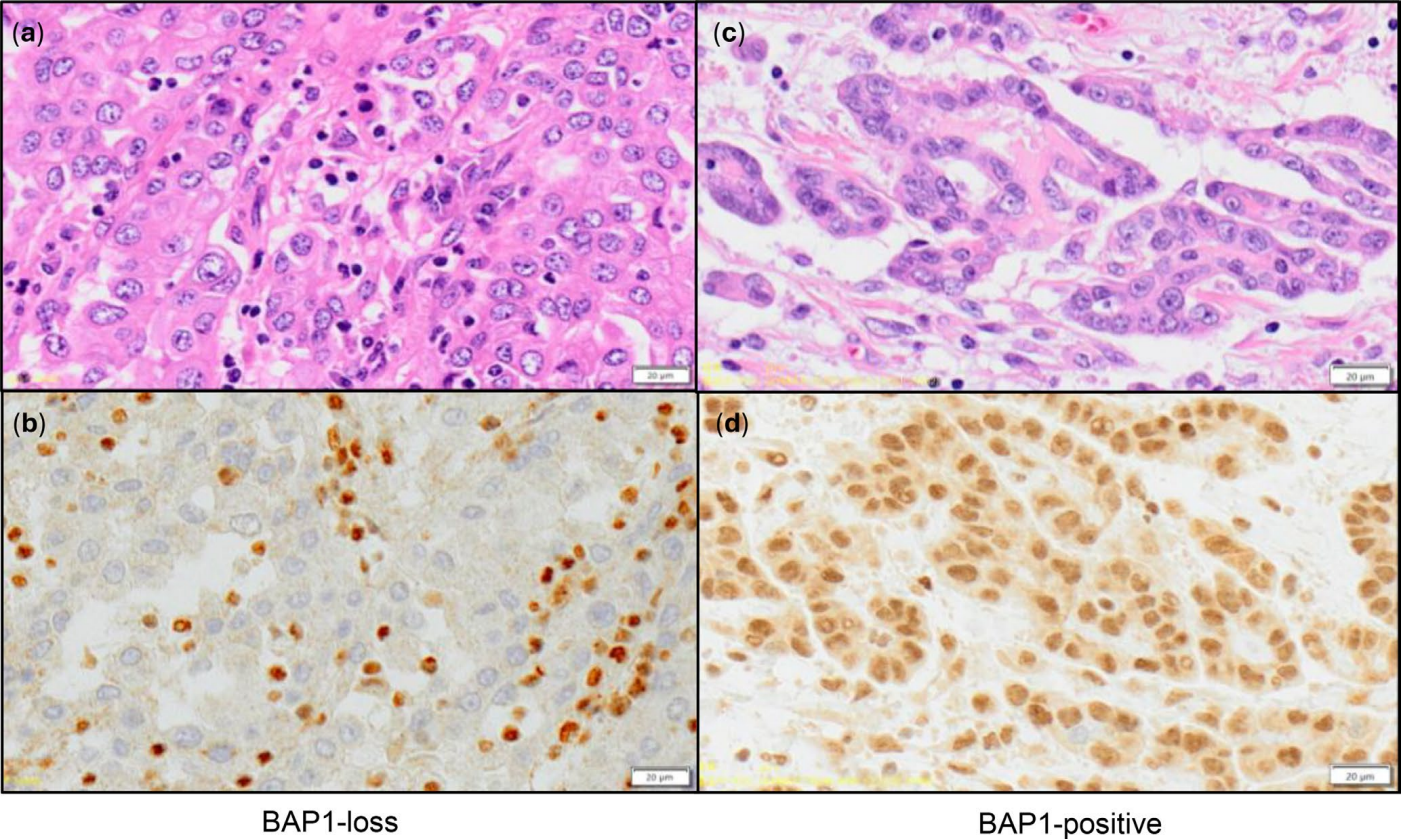
Hematoxylin and eosin (H&E) staining (a,c) and immunohistochemical staining for BAP1 (b,d) in two representative cases of BAP1‐loss and BAP1‐wild type pleural mesothelioma. Images are shown at 200 × magnification.

BAP1 status was determined by IHC. In cases with BAP1 gene alterations, BAP1 protein expression is typically lost. Moreover, even when protein expression is preserved, disruption of the nuclear localization signal can result in loss of nuclear BAP1 expression [5]. Representative IHC images of BAP1 expression are shown in Figure 2.

### 3.3 Outcomes According to BAP1 Status

**FIGURE 3:**
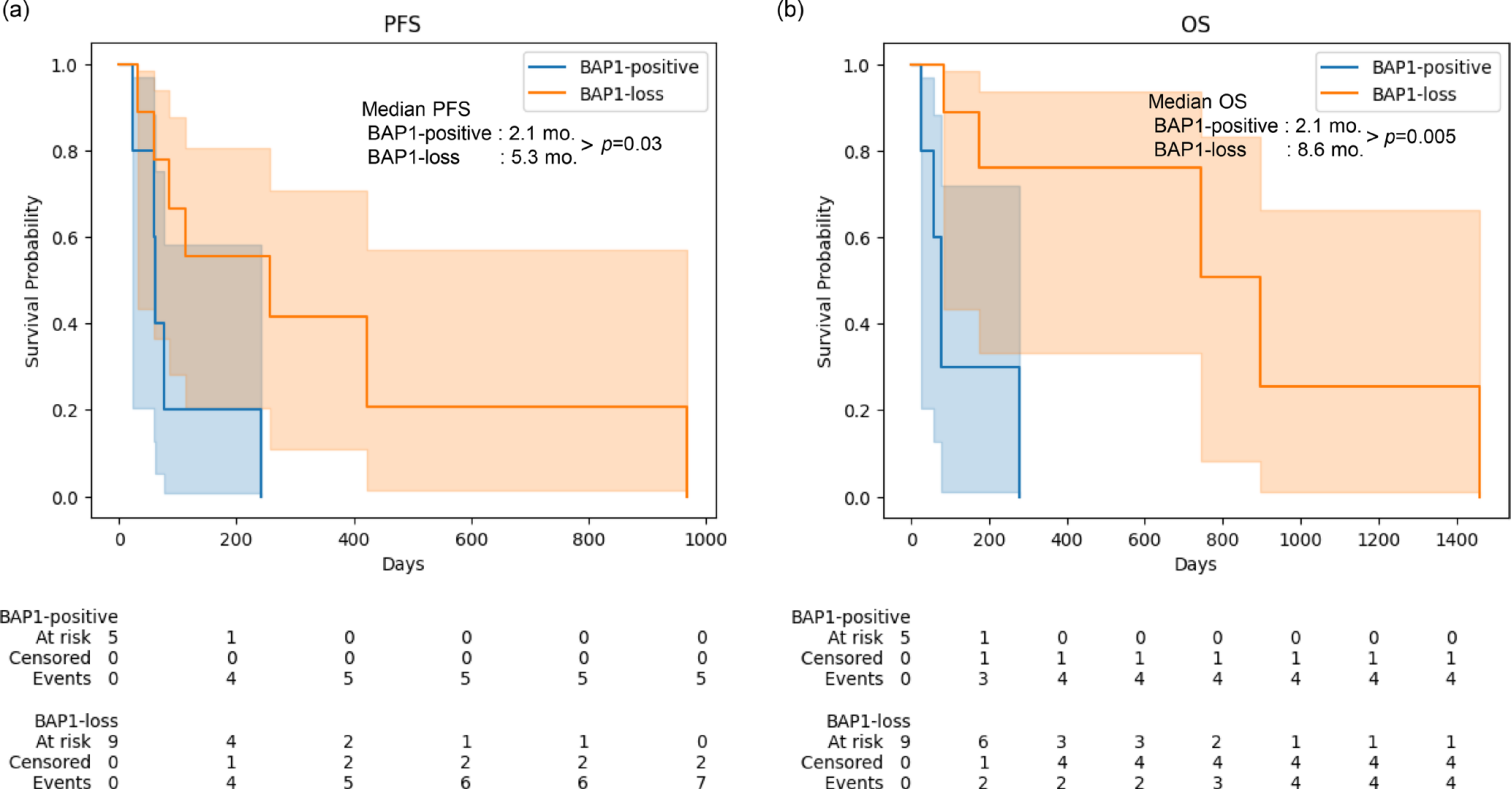
Progression‐free survival (a) and overall survival (b) according to BAP1 status.

Among patients with BAP1‐loss, three patients (33%) were continuing immunotherapy at the time of analysis, one patient (11%) discontinued treatment due to disease progression and subsequently received alternative therapy, and five patients (56%) had died. In the BAP1‐positive group, one patient (20%) received subsequent therapy after disease progression, and four (80%) had died. Median PFS was significantly longer in patients with BAP1‐loss tumors compared with those with BAP1‐positive tumors (5.3 vs. 2.1 months, respectively; *p* = 0.03; Figure 3a). RR was 37.5% with the BAP1‐loss group and 0% with the BAP1‐positive group (*p* = 0.2), while DCR was 62.5% and 40%, respectively (*p* = 0.41). Median OS was also significantly longer in the BAP1‐loss group than in the BAP1‐positive group (8.6 vs. 2.1 months, respectively; *p* = 0.005; Figure 3b). Thus, both PFS and OS were significantly prolonged in patients with BAP1 loss. Although differences in RR and DCR did not reach statistical significance, both tended to be higher in the BAP1‐loss group.

**FIGURE 4:**
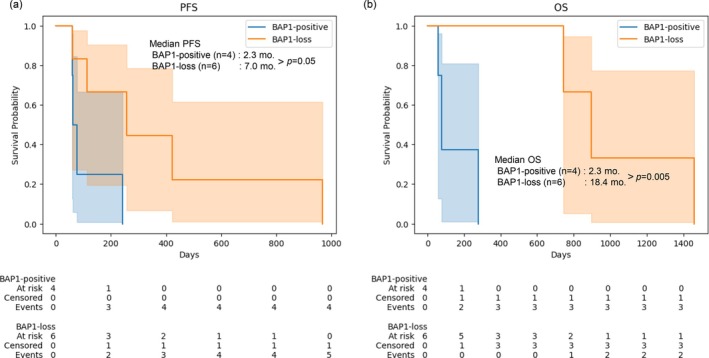
Progression‐free survival (a) and overall survival (b) in patients with epithelial pleural mesothelioma according to BAP1 status.

Subgroup analyses were performed according to histological subtype and ICI regimen. Among patients with epithelioid histology, median PFS was 7.0 months in the BAP1‐loss group (*n* = 6) and 2.3 months in the BAP1‐positive group (*n* = 4) (*p* = 0.05), while median OS was 18.4 and 2.3 months, respectively (*p* = 0.005) (Figure 4). Subgroup analysis for non‐epithelioid histology was not performed due to the limited sample size. Among the patients treated with combination therapy consisting of Ipilimumab plus nivolumab, median PFS was 7.0 months for BAP1‐loss group (*n* = 4) and 2.1 months for BAP1‐positive group (*n* = 3) (*p* = 0.06). Median OS was 8.5 and 2.1 months, respectively (*p* = 0.1). Among patients who received nivolumab monotherapy, median PFS was 2.8 months in the BAP1‐loss group (*n* = 5) and 2.3 months in the BAP1‐positive group (*n* = 2) (*p* = 0.29), while median OS was 12.1 and 2.3 months, respectively (*p* = 0.008). Although these differences did not reach statistical significance, BAP1 loss showed a stronger trend toward prolonged PFS in the Ipilimumab plus nivolumab subgroup, compared with other subgroups (Figure S1).

## 4 Discussion

In this retrospective cohort of patients with PM with immune checkpoint inhibitors, BAP1 loss was associated with prolonged PFS and OS. Median PFS was significantly longer in patients with BAP1‐loss tumors than in those with BAP1‐positive tumors (5.3 vs. 2.1 months, *p* = 0.03). Although differences in RR and DCR did not reach statistical significance, both were numerically higher in the BAP1‐loss group. These preliminary findings only provide early, tentative support for the idea that BAP1 status might serve as a potential biomarker for immunotherapy efficacy in PM. To date, only one study has directly evaluated the relationship between BAP1 status and immunotherapy outcomes in PM. Dudnik et al. [16] compared the efficacy of immunotherapy between three patients with BAP1 inactivating mutation or copy number loss and four patients without a BAP1 alteration and reported no association between BAP1 status and ICI efficacy To the best of our knowledge, no additional studies have addressed this question.

Several studies have indicated that PM patients with BAP1 loss exhibit a better prognosis compared to those with BAP1‐positive [10, 12, 13]. On the other hand, other reports have found no association with prognosis [14, 15], or have failed to confirm the correlation upon multivariate analysis [9, 11]. In this study, the favorable response to immunotherapy observed in the BAP1‐loss group might be influenced by the inherently better prognosis of this cohort. To investigate this possibility, the prognosis based on BAP1 status should ideally be compared in a patient cohort that did not receive immunotherapy. However, detailed analysis was difficult because follow‐up data were unavailable for the large number of patients. Therefore, we can't definitively conclude that BAP1 loss is a predictor of the efficacy of immunotherapy.

The biological mechanisms underlying the association between BAP1 loss and immunotherapy remain under active investigation. Xu et al. analyzed publicly available datasets, including The Cancer Genome Atlas (TCGA) and a French PM cohort, to examine the effect of BAP1 deficiency on the tumor immune microenvironment and its relationship to ICIs. Their analysis demonstrated that BAP1‐deficient tumors showed significantly increased expression of immune‐related pathways, including interferon‐α/γ signaling, as well as higher levels of activated dendritic cells, immune checkpoint receptors such as LAG3 and VISTA, chemokines, and chemokine receptors at the transcriptomic level. In addition, BAP1‐deficient tumors exhibited increased proportions of precursor exhausted T cells and M2 macrophages, along with reduced infiltration of immunosuppressive myeloid‐derived suppressor cells. A high spatial proximity between tumor cells and T cells was also observed [20]. Collectively, these findings suggest that BAP1‐deficient PM is characterized by an inflammatory tumor immune microenvironment. Similar observations have been reported in other studies examining BAP1 deficiency [18, 21, 22]. These data suggest that BAP1 status may influence the efficacy of ICI in PM. On the other hand, it has been suggested that when mutations in other genes are combined with BAP1 deficiency, different trends emerge. A preclinical study has shown that when BAP1 alterations are combined with NF2 or CDKN2A inactivation, tumor malignancy increases [23]. Furthermore, a study analyzing genetic mutations and prognosis in mesothelioma using TCGA (The Cancer Genome Atlas) data demonstrated that the co‐occurrence of BAP1 and CDKN2A mutations is associated with a poor prognosis and reduced sensitivity to chemotherapy and immunotherapy [24]. Indeed, in previous studies that attempted to develop risk assessment scores for MPM, It has been shown that survival in patients with mesothelioma does not depend on any single feature or gene alteration [25]. Based on these findings, it cannot be definitively concluded that BAP1 alone serves as a predictor of prognosis or treatment response; rather, subtype diagnosis must be performed based on multiple factors, taking into account interactions with other genes.

Subgroup analysis in our study revealed a stronger trend toward prolonged PFS in BAP1‐loss tumors treated with the combination therapy of ipilimumab and nivolumab compared with nivolumab monotherapy. Ipilimumab, an anti‐CTLA‐4 antibody, enhances T cell priming and activation. The increased infiltration and activation of immune cells in BAP1‐loss tumors, as reported in prior studies, may potentiate the efficacy of CTLA‐4 blockade, thereby contributing to the enhanced benefit observed with combination therapy.

Our study has several limitations. First, its retrospective design introduces the potential for selection bias. Second, PM is a rare tumor with a small sample size, and the exclusion of many patients may have led to selection bias. We compared the 14 patients included in the study with the 9 patients who were excluded due to insufficient tissue samples; there were no significant differences in patient characteristics (Table S2). Although this study observed a statistically significant difference in the efficacy of immunotherapy based on BAP1 status, validation in a larger cohort is necessary. Third, ICI was administered after disease recurrence; in several cases, it was used as first‐line systemic therapy in the recurrent setting, the effects of prior chemotherapy were not considered. Because all patients had previously undergone chemotherapy including adjuvant therapy, the tumor microenvironment may have been altered, potentially influencing the efficacy of ICIs. Fourth, while this study analyzed BAP1 status using IHC alone, discrepancies may exist with the results of genetic analysis. In fact, a study comparing BAP1 gene analysis using NGS with BAP1 protein expression assessed by IHC found discrepancies in 14% of cases [26]. If results from genetic analysis could also be included in the analysis as needed, more accurate data could likely be obtained. Finally, we did not directly analyze immune cell infiltration or other features of the tumor microenvironment in our cohort. To identify predictive biomarkers for immunotherapy in PM, future studies incorporating detailed immunophenotyping and larger prospective patient populations are needed.

This study examined how BAP1 status affects the effectiveness of immunotherapy. BAP1 plays a crucial role in mesothelioma diagnosis and has been suggested as a predictor of prognosis and treatment response. Additionally, recent studies have evaluated the efficacy of enhancer of zeste homolog 2 (EZH2) targeted therapies [27, 28, 29] and poly ADP ribose polymerase (PARP) inhibitors [30] for treating BAP1‐loss mesothelioma. If these agents are confirmed to be effective, treatment options may vary depending on BAP1 status. It is expected that the clinical application of BAP1 will be advanced by future research. In conclusion, a molecular profile that includes BAP1 deletion may serve as a biomarker for predicting response to immunotherapy in PM. Prospective validation in larger cohorts is warranted.

## Author Contributions

**Hironori Ishibashi:** resources, writing – review and editing. **Yasunari Miyazaki:** writing – review and editing, supervision, funding acquisition. **Rie Sakakibara:** conceptualization, resources, validation, writing – review and editing, project administration, funding acquisition. **Susumu Kirimura:** resources, writing – review and editing, visualization. **Kenichi Okubo:** resources, writing – review and editing. **Reina Imase:** methodology, formal analysis, investigation, data curation, writing – original draft, visualization.

## Funding

This work was supported in part by Grants‐in‐Aid for Scientific Research from the Ministry of Education, Culture, Sports, Science and Technology, Japan, including JSPS KAKENHI (JP25K19460).

## Ethics Statement

This is a retrospective observational study. This study has been approved by the Ethics Review Committee of Institute of Science Tokyo (Approval No. M2020‐224), and informed consent was obtained from all patients.

## Conflicts of Interest

Yasunari Miyazaki received honoraria for lectures from Bristol Myer Squibb, Ono Pharma Co. Ltd. and Eli Lilly Japan K.K. Rie Sakakibara received honoraria for lectures from Ono Pharma Co. Ltd. The other authors declare no conflicts of interest regarding this manuscript.

## Supporting information


**Figure S1:** Progression‐free survival in patients treated with ipilimumab plus nivolumab and nivolumab monotherapy according to BAP1 status.

**Table S1:** Patient characteristics.

**Table S2:** Comparison of patients characteristics in the included patients and excluded patients.

## Data Availability

The datasets generated and/or analyzed during the current study are not publicly available due to ethical and institutional restrictions related to patient confidentiality but are available from the corresponding author on reasonable request, subject to approval by the institutional review board.
