# Supplementary material for: BAP1 Loss Might Be a Predictive Biomarker for Immunotherapy Response in Pleural Mesothelioma
Source: Thorac Cancer. 2026 Jul 1;17(13):e70343. doi: 10.1111/1759-7714.70343 (PMC13321164; doi:10.1111/1759-7714.70343)
Supplement: Supplementary file 1 — Figure S1: Progression‐free survival in patients treated with ipilimumab plus nivolumab and nivolumab monotherapy according to BAP1 status. [file TCA-17-e70343-s002.pdf]

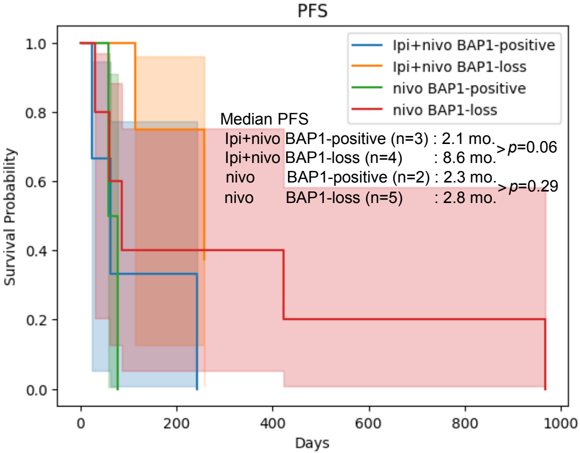

**Ipi+nivo BAP1-positive**						
At risk	3	1	0	0	0	0
Censored	0	0	0	0	0	0
Events	0	2	3	3	3	3
**Ipi+nivo BAP1-loss**						
At risk	4	2	0	0	0	0
Censored	0	1	2	2	2	2
Events	0	1	2	2	2	2
**nivo BAP1-positive**						
At risk	2	0	0	0	0	0
Censored	0	0	0	0	0	0
Events	0	2	2	2	2	2
**nivo BAP1-loss**						
At risk	5	2	2	1	1	0
Censored	0	0	0	0	0	0
Events	0	3	3	4	4	5
