# Supplementary material for: BAP1 Loss Might Be a Predictive Biomarker for Immunotherapy Response in Pleural Mesothelioma
Source: Thorac Cancer. 2026 Jul 1;17(13):e70343. doi: 10.1111/1759-7714.70343 (PMC13321164; doi:10.1111/1759-7714.70343)
Supplement: Supplementary file 2 — Table S1: Patient characteristics. [file TCA-17-e70343-s001.docx]

Table S1. Patient characteristics

| Patient number | Histology | BAP1 status | Surgery | Adjuvant therapy | Time to  recurrence (months) | Prior systemic therapy before ICI | Treatment sequence | ICI regimen | Setting of ICI  (recurrent/  unresectable) | Line of  therapy  (recurrent setting) | Line of  therapy  (overall, including adjuvant) | Best response | PFS  (months) | OS  (months) |
| --- | --- | --- | --- | --- | --- | --- | --- | --- | --- | --- | --- | --- | --- | --- |
| 1 | Epithelioid | Loss | Yes | Plutinum + pemetrexed | 26 | None | Surgery→Adjuvant CT  →Recurrence→ICI | Nivolumab | Recurrent | 1st | 2nd | PR | 32.2 | 48.7 |
| 2 | Epithelioid | Positive | Yes | Plutinum + pemetrexed | 12.6 | None | Surgery→Adjuvant CT  →Recurrence→ICI | Nivolumab | Recurrent | 1st | 2nd | SD | 2.6 | 2.6 |
| 3 | Epithelioid | Loss | Yes | Plutinum + pemetrexed | 22 | None | Surgery→Adjuvant CT  →Recurrence→ICI | Nivolumab | Recurrent | 1st | 2nd | PD | 2 | 29.9 |
| 4 | Biphasic | Loss | Yes | Plutinum + pemetrexed | 5.9 | None | Surgery→Adjuvant CT  →Recurrence→ICI | Nivolumab | Recurrent | 1st | 2nd | PD | 1 | 5.8 |
| 5 | Biphasic | Loss | Yes | Plutinum + Gemcitabine | 10.5 | Plutinum + pemetrexed | Surgery→Adjuvant CT  →Recurrence→CT→ICI | Nivolumab | Recurrent | 2nd | 3rd | PD | 2.8 | 2.8 |
| 6 | Epithelioid | Loss | No | None | NA | Plutinum + pemetrexed | CT→PD→ICI | Ipilimumab + Nivolumab | Unresectable | 2nd | 2nd | SD | 8.6 | 24.8 |
| 7 | Epithelioid | Loss | Yes | Plutinum + pemetrexed | 15.5 | None | Surgery→Adjuvant CT  →Recurrence→ICI | Nivolumab | Recurrent | 1st | 2nd | PD | 14.1 | 12.1 |
| 8 | Epithelioid | Positive | Yes | Plutinum + pemetrexed | 4.1 | None | Surgery→Adjuvant CT  →Recurrence→ICI | Ipilimumab + Nivolumab | Recurrent | 1st | 2nd | SD | 8.1 | 9.2 |
| 9 | Epithelioid | Positive | Yes | Plutinum + pemetrexed | 18.5 | None | Surgery→Adjuvant CT  →Recurrence→ICI | Ipilimumab + Nivolumab | Recurrent | 1st | 2nd | PD | 2.1 | 2.1 |
| 10 | Sarcomatoid | Positive | Yes | None | 0.9 | Plutinum + pemetrexed  as neoadjuvant  therapy | Neoadjuvant CT  →Surgery→Recurrence  →ICI | Ipilimumab + Nivolumab | Recurrent | 1st | 2nd | PD | 0.8 | 0.8 |
| 11 | Epithelioid | Loss | Yes | Plutinum + pemetrexed | 18.2 | None | Surgery→Adjuvant CT  →Recurrence→ICI | Ipilimumab + Nivolumab | Recurrent | 1st | 2nd | PR | 5.3 | 5.3 |
| 12 | Epithelioid | Loss | Yes | Plutinum + pemetrexed | 11.7 | None | Surgery→Adjuvant CT  →Recurrence→ICI | Ipilimumab + Nivolumab | Recurrent | 1st | 2nd | PR | 3.8 | 8.3 |
| 13 | Epithelioid | Positive | No | None | NA | Pemetrexed | CT→PD→ICI | Nivolumab | Unresectable | 2nd | 2nd | PD | 2 | 2 |
| 14 | Biphasic | Loss | Yes | None | 3.5 | Plutinum + pemetrexed  as neoadjuvant  therapy | Neoadjuvant CT→Surgery  →Recurrence→ICI | Ipilimumab + Nivolumab | Recurrent | 1st | 2nd | SD | 8.6 | 8.6 |

CT, chemotherapy; ICI, immune checkpoint inhibitor; PFS, progression free survival; OS, overall survival; NA, not applicable.
