# Supplementary material for: BAP1 Loss Might Be a Predictive Biomarker for Immunotherapy Response in Pleural Mesothelioma
Source: Thorac Cancer. 2026 Jul 1;17(13):e70343. doi: 10.1111/1759-7714.70343 (PMC13321164; doi:10.1111/1759-7714.70343)
Supplement: Supplementary file 3 — Table S2: Comparison of patients characteristics in the included patients and excluded patients. [file TCA-17-e70343-s003.docx]

Table S2. Comparison of patients characteristics in the included patients and excluded patients

|  | **Total**  **(n=23)** | **Included**  **(n=14)** | **excluded**  **(n=9)** | ***p*-value** |
| --- | --- | --- | --- | --- |
| Median age (range) | 64 (45–80) | 63.5 (45–80) | 69 (55–79) | 0.63 |
| Sex  Male  Female | 21 (91)  2 (9) | 13 (93)  1 (7) | 8 (89)  1 (11) | 0.74 |
| Histological type  Epithelial type  Biphasic  Sarcoma type | 18 (78)  4 (17)  1 (4) | 10 (71)  3 (21)  1 (7) | 8 (89)  1 (11)  0 | 0.55 |
| Asbestos exposure | 16 (70) | 10 (71) | 6 (67) | 0.81 |
| Smoking history | 18 (78) | 11 (79) | 7 (78) | 0.96 |
| ECOG PS  0–1  2–4 | 21 (91)  2 (9) | 13 (93)  1 (7) | 8 (89)  1 (11) | 0.74 |
| Stage  　Ⅰ–Ⅱ  　Ⅲ–Ⅳ | 15 (65)  8 (35) | 10 (71)  4 (29) | 5 (56)  4 (44) | 0.44 |
| Post surgery | 19 (83) | 12 (86) | 7 (78) | 0.62 |
| Pretreatment  (including adjuvant therapy)  Platinum+Pemetrexed  Platinum+Gemcitabine  Pemetrexed  Vinorelbine | 20 (71)  5 (18)  1 (7)  2 (4) | 13 (87)  1 (7)  1 (7)  0 | 7 (54)  4 (31)  0  2 (15) | 0.09 |
| ICI  Ipilimumab+Nivolumab  Nivolumab | 9 (39)  14 (61) | 7 (50)  7 (50) | 2 (22)  7 (78) | 0.18 |

Data are presented as number (%) unless otherwise specified. *P*-value was calculated using the Mann–Whitney U test for continuous variables and chi-square test or Fisher's exact test for categorical variables, as appropriate.

ECOG PS, Eastern Cooperative Oncology Group Performance Status; ICI, immune checkpoint inhibitor.
